# The impact of clinical and translational research on the quality of life during the metastatic colorectal cancer patient journey

**DOI:** 10.3389/fonc.2023.1272561

**Published:** 2023-10-16

**Authors:** Marta Rodriguez Castells, Iosune Baraibar, Javier Ros, Nadia Saoudi, Francesc Salvà, Ariadna García, Adriana Alcaraz, Josep Tabernero, Elena Élez

**Affiliations:** ^1^ Medical Oncology Department, Vall d’Hebron University Hospital, Barcelona, Spain; ^2^ Vall d’Hebron Institute of Oncology (VHIO), Barcelona, Spain

**Keywords:** patient journey, quality of life, colorectal cancer, patient reported outcomes, patient reported outcome measures

## Abstract

The journey of metastatic colorectal cancer patients is complex and challenging, requiring coordination and collaboration between multiple healthcare providers. Understanding patients’ needs, fears, feelings, concerns, and behaviors is essential for providing individualized patient-centered care. In recent years, mCRC patients have experienced improvements in clinical outcomes, from 16 months of overall survival to 32 months, thanks to research. However, there is still room for improvement, and integrating clinical and translational research into routine practice can help patients benefit from treatments and techniques that would not be an option. In the Journey of mCRC patients, living well with cancer and quality of life becomes a priority given the outcomes of the disease. Patient reported outcomes (PRO) and Patient Reported Outcome Measures (PROMs) are becoming therefore new estimands in Oncology. Patient advocates represent important figures in this process by prioritizing issues and research questions; evaluating research designs and the performance of the research; the analysis and interpretation of data; and how results are disseminated. Multidisciplinary Tumor Boards and shared decision-making is essential for designing treatment strategies for individual patients. Quality of Life is often prioritized only when it comes to refractory advanced disease and end-of-life care, but it has to be integrated from the beginning, as the emotional impact of diagnosis leads to a vulnerable situation where patients’ needs and preferences can be easily overseen. First-line treatment will be chosen among more treatment options than subsequent lines, with longer progression-free survival and a bigger impact on the outcomes. Practicing patient-centered care and optimizing first-line treatment for colorectal cancer patients requires a comprehensive understanding of patient experience and treatment outcomes, which can guide clinical practice and inform regulatory decisions for the benefit of patients.

## 1 Introduction

**Figure 1:**
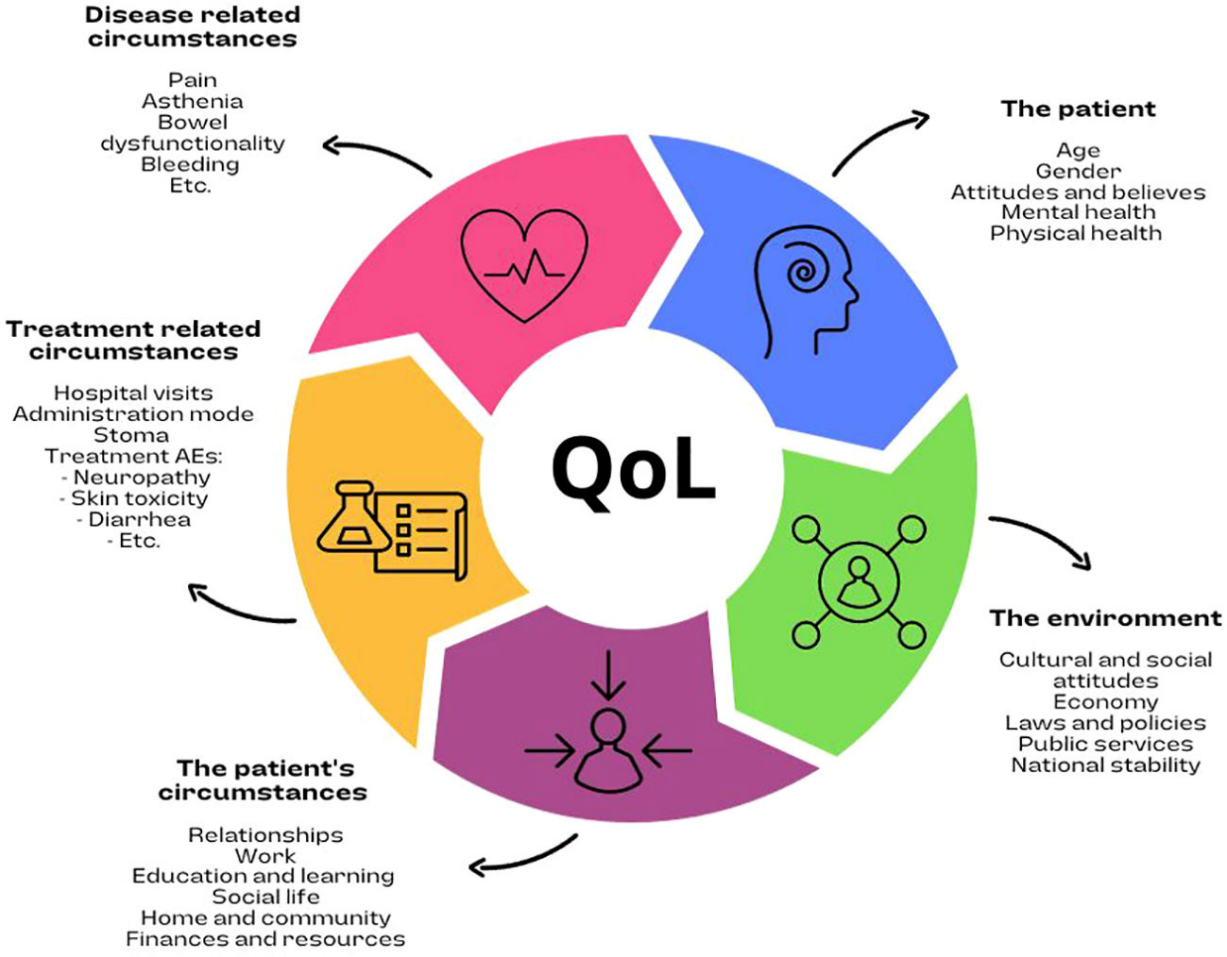
Factors that influence the patient’s quality of life.

Colorectal cancer (CRC) is the third leading cause of cancer deaths worldwide. In 2020, approximately two million new CRC cases were diagnosed, and one million CRC patients died (1). The global burden is increasing every year, attributed to the adoption of Western lifestyles, and this increase is expected to increase up to 2.2 million new cases and 1.1 million deaths by the year 2030 (2). The journey of metastatic colorectal cancer (mCRC) patients is a complex and challenging experience, requiring coordination and collaboration between multiple healthcare providers, starting with diagnosis, followed by different treatment and management options (3). Multidisciplinary tumor boards, composed of physicians from every specialty involved in the management of patients with CRC, including molecular tumor boards, guide the entire process (4, 5). Although tumor boards can provide the best advice, we cannot oversee patients’ needs and individual circumstances as well as particular tumor features, which are essential for providing individualized patient centered care. There are many factors that influence the patient’s quality of life (QoL) and affect the process of shared decision-making when practicing patient-centered care (**Figure 1**). Understanding the patient’s needs, fears, feelings, concerns, and behaviors will help the community and healthcare providers enhance the experience of the mCRC patient journey. A journey where QoL becomes a priority given the outcomes of the disease.

In the last few years, mCRC patients have experienced an enhancement in their clinical outcomes from approximately 16 months of overall survival (OS) to 32 months (6), provided by the development of new drugs that have been added to the continuum of care based on the new evidence derived from large phase III trials (7–10). Regardless of these optimistic results, there is still room for improvement. Unfortunately, the implementation of drugs and the updating of guidelines have a delay of years, and healthcare systems have inherent deficiencies that make the introduction of novel strategies into the clinic difficult (11). Furthermore, although clinical outcomes such as OS and progression free survival (PFS) are obviously important, we are far from our patients’ expectations and wishes; hence, QoL must be prioritized and patients need to be involved in treatment-decision making (12). Integrating clinical and translational research into routine practice might help patients benefit from treatments and techniques that otherwise wouldn’t be an option, while also helping generate evidence-based medicine. In this setting, having trained and empowered patients that understand the importance of this research is fundamental to improving the patient’s experience and ensuring smooth bidirectional communication (13). Patient advocates can help make these processes easier as trained and expert patients (14).

Clinical research even in upfront treatment options, are interesting for selected patients. When it comes to translational research, this approach gives clinical scientists the opportunity to gain knowledge about the patient’s tumor, while the patient could obtain a personalized approach that otherwise probably could not be performed. Translational research has an impact on the patient journey by translating findings from the laboratory into effective clinical interventions and providing the opportunity to understand the underlying biology of colorectal cancer, allowing for a more precise classification of the disease, and identifying potential therapeutic targets (15, 16). Most likely, in the near future, this classification will integrate comprehensive knowledge about gene alterations, tumor microenvironment, host immunity characteristics, and protein expression profiling of each case, which will be followed along the patient journey, helping physicians select the most appropriate and personalized sequence of treatments.

**Figure 2:**
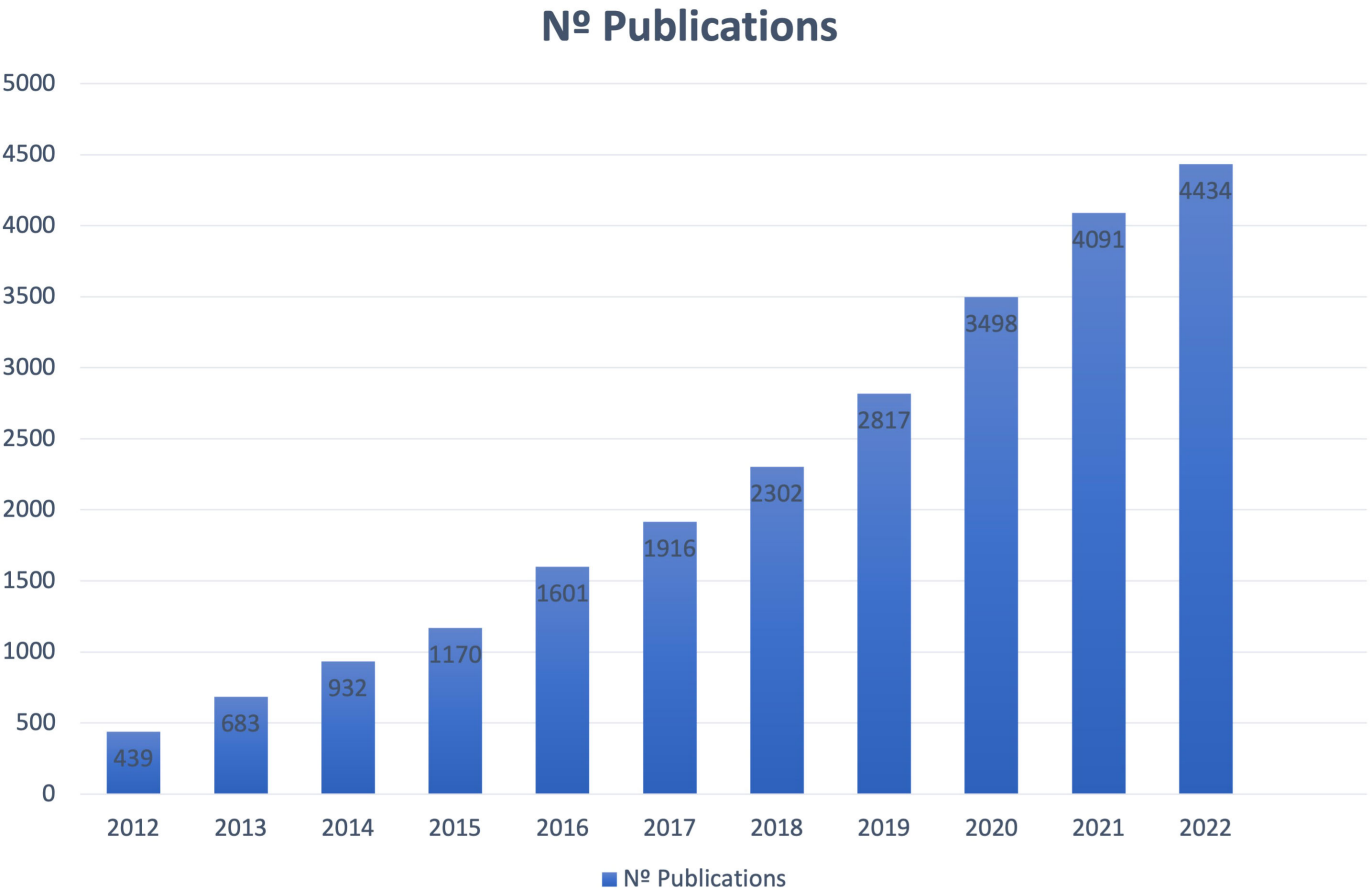
Number of publications for the search of the term “Patient Reported Outcome” in PubMed database.

Any self-report of the status of a patient’s health condition that comes directly from the patient without interpretation of the patient’s response by a clinician or anyone else that might introduce bias is considered a patient reported outcome (PRO) (17). Although slowly, the medical community is fortunately shifting towards a patient centered model, where QoL assessments by means of PRO are becoming new estimands in oncology (17–22). The growing interest of physicians in this field can be proven by a simple literature search in PubMed for PRO, which throws up ten times more results than ten years ago in all fields of healthcare (**Figure 2**).

As the shift away from the old physician-centered care model and toward a patient-focused approach continues to grow, the goal of the next few pages is to look at the most important aspects of QoL from the time of diagnosis until the choice of the first treatment for patients with mCRC, as the first experience with the disease will probably shape the rest of the patient’s journey. QoL is frequently prioritized only when it comes to refractory disease and end-of-life care, but it should be a priority from the beginning since the emotional effect of diagnosis creates a vulnerable scenario where patients’ needs and preferences can be easily overlooked. Additionally, first-line treatment will be selected from a wider range of possibilities than subsequent lines, and it will have a greater influence on the outcomes and longer progression-free survival (23). Getting to know patients’ preferences and discussing the advantages and disadvantages of the treatment when designing the treatment plan is crucial to practice patient-centered care and defining the doctor-patient relationship.

## 2 Assessing and reporting quality of life

Thinking about the needs that the patient has as an individual and trying to meet those needs and ensure quality of life for the patients while they are dealing with this disease is crucial to practicing patient-centered care, as they don’t want this disease to dominate every aspect of their lives. Matching the right treatment to an individual patient can be done by personalizing in many ways, like using precision oncology medicine and molecular profiling, but it can also be done by choosing a treatment based on patient preferences, needs, values, and obligations, as well as other considerations. There is a need for improved communication about what is important to a patient and ensuring that the patient understands what is important to the clinical team. Making those messages line up as we choose the right treatment for an individual patient will allow us to successfully practice patient-centered care and improve outcomes. Those outcomes are often disease-based (disease control type outcomes: PFS, overall response rate (ORR), etc.), but they also include outcomes like maintaining QoL and maintaining the patient’s functionality and ability to engage in the rest of their lives beyond cancer.

To improve it, we need to be able to measure it and understand its components, so we can focus on them and characterize them clearly. QoL is not affected by adverse events alone. It includes adverse events and patient-reported outcome measurements (PROMs), as well as other factors. Adverse events are treatment-related and classically assessed by the Common Terminology Criteria for Adverse Events (CTCAE) to ensure we are keeping the patient safe (24). They can be symptomatic, but sometimes they are also asymptomatic and do not affect the patient on a day-to-day basis, such as laboratory abnormalities. PROMs, on the other hand, are clinically meaningful for patients; they are measurable by patients and reported by patients (25). To overcome these limitations, a new classification of Patient-Reported Outcomes Version of the Common Terminology Criteria for Adverse Events (PRO-CTCAE) has been designed, that could be used across the therapeutic development process to address important questions related to the tolerability profile of a specific therapy (26).

While the clinical team is best suited to report some objective toxicities and laboratory findings, patients themselves are best suited to report subjective experiences. These are symptoms and functional scales (satisfaction with clinical care, adherence to medication, perceived value of treatment). In addition, prior research points out that symptoms and the severity of them are often underestimated by the clinical team, leading to discrepancies between patient self-reporting and clinicians (27). There are other factors that are also affecting the patient but that are usually never measured, like the number of hospital visits, their ability to work or travel, anxiety, financial concerns, burden on family, medication constraints, etc.

Several PROMs and scales are available for assessing health-related quality of life (HRQoL) in patients with cancer. The Functional Assessment of Cancer Therapy-General (FACT-G) and the European Organization for Research and Treatment of Cancer Quality of Life Questionnaire (EORTC QLQ-C30) are two of the most commonly used PROMs (28). These questionnaires include multidimensional assessments with items pertaining to physical, functional, emotional, and social well-being.

Other PROMs designed specifically for patients with mCRC include the EORTC QLQ-Colorectal 29 (EORTC QLQ-CR29) (29) and the Functional Assessment of Cancer Therapy-Colorectal (FACT-C) (30). Both questionnaires include specific questions about bowel function, ostomy-related aspects, and assessing sexual functionality. The EORTC QLQ-Colorectal Liver Metastases module (EORTC QLQ-LMC21) is meant to find out how patients feel about their colorectal metastases’ treatment (31). These instruments have been developed, validated, and assessed within the population we want to study.

These questionnaires do not include questions related to common concerns in colorectal cancer patients, such as QoL related to skin toxicity, peripheral neuropathy, etc. For this purpose, other general questionnaires can be used to assess different items: peripheral neuropathy (FACT/GOG-Ntx25) (32), depression/anxiety (PHQ-926, HADS27) (33, 34), general QoL (FS-36, EQ-5D29) (35, 36), pain (BPI30) (37), fatigue (FACIT- Fatigue31, BFI31) (38), hand food syndrome (HFS-14) (39), Dermatology Life Quality Index (DLQI) (40), etc.

PROMs in colorectal cancer randomized clinical trials between 2014 and 2019 have exponentially increased, and in this population, EORTC and 5Q-5D questionnaires have been the most used for evaluating QoL (41). In routine clinical cancer care, real world data are starting to emerge, but implementation in healthcare systems not related to research is still not standardized. There are several guidelines for developing and evaluating PROMs (41). The first recommendation is to define the meaningful outcomes for the colorectal cancer patient’s journey, representing their values and needs. Administration modes include paper tools, remote monitoring electronic tools, etc. that can be chosen after evaluation of the population to study. As patients can sometimes experience access barriers (internet access, electronic devices, etc.), in particular cases, using paper questionnaires can help reduce under-reporting when provided at medical visits. It is important to avoid patient burden and increase completion rates that the number of asked items is carefully considered, as missing reports represent a limitation when it comes to interpreting the data (20, 41). Other main constraints to PROMs are interpretation and liability issues as well as lack of resources/funding as major barriers for PRO implementation (42). There are no specific recommendations about the frequency with which patients should fill out the PROMs; it depends on the feasibility and the population that is being studied.

We should not just measure a number from these surveys but understand what those numbers mean if we want to comprehend the patient’s trajectory in terms of QoL over time or compare treatments to each other. Even though not systematically used, a useful tool for this purpose is the Minimally important difference (MID) or clinically meaningful change, which represents the amount of change in a given PROM that patients perceive as important, leading the patient or clinician to consider a change in management.

Not only understanding, defining, and assessing PRO is important, but analyzing and reporting them properly is also needed. In our patient-centered era, where precision medicine and personalized treatment is re-defining how we treat patients, defining outcome/cost is crucial for patients, clinicians, and regulatory agencies. For that purpose, QoL data and, therefore, PROMs are essential. The importance of capturing and reporting HRQoL in clinical trials has been increasingly recognized and strategies to improve them have been implemented. As an example, regarding the CONSORT (Consolidated Standards of Reporting Trials) Statement commonly used to improve the reporting of randomized controlled trials (that lacks guidance on the reporting PRO) was updated in 2013 with the development of the CONSORT PRO extension, in which PROs are primary or important secondary end points. Improved reporting of PRO data should facilitate robust interpretation of the results from RCTs and inform patient care. Guidelines for the inclusion of PROs in clinical trials have also been developed by the SPIRIT-PRO extension (43).

Another initiative comes from the National Cancer Institute (NCI) by providing founding support for correlative HRQoL studies. Even tough, a study reported that the publication rate of NCI-supported trials in peer-review journals was 54%, and of these, only 62% had a published HRQoL result. 45% of the published HRQoL endpoints results were in the main publication (44). As some authors describe, there are several limitations that are making it difficult to get high quality description and analysis of HRQoL. First of all, even if the “single publication” approach enables authors to link HRQoL and clinical endpoints together, frequently HRQoL results are only minimally described, if described, due to limited journal space. A second limitation is related to the authorship list, as the lead HRQoL author must also find a place in it. Parallel papers reporting on the same trial could address both limitations, but again journal space and editorial position is that only one paper is of interest to the journal (45).

Across all oncology, targeted therapy, and tumor agnostic treatments in the scenario of precision oncology are resulting in improved outcomes, not only limited to survival and other surrogates, but also QoL (46, 47). But when it comes to some types of precision medicine trials like early phases or complex newer designs of clinical trials (ex. basket trials, umbrella trials, master observational protocols) and early translational research under the precision oncology paradigm, validated PRO questionnaires might not be the best solution, since precision medicine trials are different to randomized large phase 3 trials where these scales have been validated. They tend to have a smaller number of patients included and use single-arm tests for detecting efficacy signals among patients who share a common molecular profile. Patient’s perspective needs still to be included but will likely be more a kind of discovery description than of formal hypothesis testing. Thus, some authors conclude that traditional PRO endpoints are not the most appropriate in this setting, basically because they are providing an early look at the patient experience of new drugs or combination of them, which cannot be summarized in conventional endpoints and, on the other hand, there will probably be a lack of statistical power that difficult the trial to throw conclusions (48). There is a growing need of real-world-data that emerges of molecular testing, as each molecular subset of mCRC is more precisely characterized, leading to difficulties to perform large clinical trials. This strategy could enhance understanding about predictive biomarkers and resistance mechanisms of less common molecular subtypes. By integrating molecular features, clinical outcomes and QoL data in high-quality shared international databases, we will allow to include the patient experience into the risk-benefit equation of the patient journey. Additionally, some differences in QoL between the real-world-data and clinical trials results across countries might be detected, as a survey suggests (49).

## 3 Patient advocacy

Overall survival in mCRC has improved in the past decade from approximately 16 to 32 months (6). Patient participation in clinical trials has been crucial to improving these outcomes. Science needs involved and committed patients to continue developing the best strategy to overcome this disease, and the best way to achieve this development is through patient education and empowerment. Emerging strategies include training programs for patient advocates, a sort of “patient school,” to strengthen patient involvement in research, health policy, and healthcare. Organizations like the British Medical Journal (BMJ) (50), Fight CRC (51), Facing Hereditary Cancer Empowered (FORCE) (52), Friends of Cancer Research (FOCR) (53), Patient-Centered Outcomes Research Institute (PCORI) (54), and the Research Advocacy Network (55), among others, have established this type of advocacy training program. Patient empowerment and patient advocacy are crucial to helping patients understand how valuable their collaboration is. Aspects of empowerment include health literacy, shared decision-making, and self-management. Currently, there is a need for a standardized set of core competencies within colorectal cancer research advocacy, as this is important to ensure patients are receiving training that benefits their learning experience and ultimately their influence and impact when working with researchers. However, on the other hand, researchers have the duty to meet some social conditions: transparency, trust, respect, and involvement (16). The plurality and diversity of patients’ needs need attention. Although proven challenging, the participation of patients or their representatives in every area of research, including basic, translational, and clinical research, can influence the agenda setting. Patient advocates help by prioritizing issues and research questions; evaluating research designs and the performance of the research; the analysis and interpretation of data; and how results are disseminated (56).

There is not much information about how mCRC patients feel while taking part in clinical trials and translational research initiatives, but a general survey reveals that the majority of patients are willing to take part in clinical trials, primarily because it might improve their own treatment, but many of them are also interested in helping others and making a contribution to scientific research (57). In the setting of early translational research requiring retrieval of samples (biopsies, either solid or liquid) under the precision oncology paradigm, it is fundamental that the process of informed consent is performed properly and patient advocacy could help in that process. Little is known about how patients feel about participating in trials requiring these types of samples or their QoL. The major concern arises when mandatory biopsies are required in clinical trials as part of translational research as this fact raises ethical concerns that relate to the risk of harm to participants, the adequacy of performed voluntary informed consent, and the potential misunderstanding among research participants about the benefit they could obtain of this process. In fact, a survey was performed in Phase I clinical trial participants showing that almost two-thirds of them misunderstood the lack of personal benefit of a research biopsy. Regarding risk assessment, results were positive, as patients were very confident that the benefits of research biopsies outweighed the risks and that the potential knowledge to be gained from that procedure was important. Biopsies were not seen as harmful or frightening, albeit somewhat painful, and less than a fifth would decline a trial for having a mandatory biopsy (58).

Translational research is now being promoted as an explicit strategy to overcome or at least reduce gaps and bottlenecks in research and can potentially help reduce the slowness of scientific advances. Policy makers and funding organizations have argued for the importance, potential, and promise of translational research, which has resulted in several funding programs, journals, research centers, and educational programs devoted to translational research. This approach can only be done with the collaboration of patients, who accept being subjected to additional blood (or other fluid) extraction, biopsies, genetic testing, questionnaires, etc. Occasionally, the patient itself could obtain direct benefit from this research. However, on many occasions, relevant questions such as how benefit is defined, for whom, and by whom are avoided (16).

All the research procedures have an impact on the patient’s journey. On the one hand, all fields of research often require more hospital visits, blood extractions, etc. But on the other hand, it could have a positive impact too, as they could feel more closely monitored, actively participate in scientific development, and, in some cases, receive a treatment that will take years to be considered standard of care. With the new drugs coming in the next few years, there is an increasing need to adapt the estimands in research and clinical trials to PRO and focus on quality data about QoL, as they are important for patients with mCRC when making treatment choices and for regulatory agencies approving drug therapies. An interesting approach proposed by Perrone et al. could be a model of patient-journey studies (PJS), where patients are followed from diagnosis across subsequent treatment lines, adapting to every new drug that arrives in the practice. PJS could significantly optimize the treatment of patients in clinical practice and could reflect the whole story of our patients’ journeys (59).

## 4 Shared decision making

Designing a treatment strategy for each individual patient is a complex process. There are many aspects to consider, and many figures will be involved. Starting with the patient itself and continuing with the clinical team and other crucial figures that will string along with the patient during their journey, such as psychologists, nutritionists, research nurses, pharmacists, trial coordinators, geriatric oncologists, administrators, primary care professionals, social services, physiotherapists, etc. Coordination and collaboration between all of them by creating a model of patient-centered care where all these figures can work together will enhance the journey of oncologic patients. This approach can be performed in many cases by Multidisciplinary Tumor Boards (MDTB). MDTB are key in defining the best treatment strategy, and each case is individualized by preferences, comorbidity risk, and treatment options. Their recommendations can change overall management decisions in colorectal cancer in 6–29% of the cases, resulting in a weighted average change of 16.2% (60).

Treatment options are carefully revised by international consensus guidelines (61, 62) that provide the best advice in general terms, but clinical teams’ duty is to evaluate and personalize treatment to achieve a patient-centered care where shared decision making becomes a reality. This personalized treatment plan may include a full understanding of the clinical and molecular features of the tumor, radical treatment options in some cases with or without adjuvant or neoadjuvant strategies, treatments to control symptoms (such as obstruction, pain, etc.), or systemic first-line treatment, which may include enrollment in clinical trials in some cases. It is crucial to take the time to explain to the patient what has led to the design of their specific treatment plan. In fact, cancer patients request information on not only survival estimands but also HRQoL (63). As the doctor-patient relationship is being built, listening to the patient, and answering all these questions will ensure shared decision making in front line settings, respecting patients’ autonomy while adapting the strategy to their needs, wishes, and values.

### 4.1 Optimizing the first-line treatment in mCRC patients

In colorectal cancer care, as across all of oncology, patient-centeredness is considered a component of high-quality care (64). In this context, there is increasing interest in understanding the patient experience. Therefore, assessing the impacts of both the treatment and the disease on HRQoL is an urgent need. PROs can bridge the gap in reported HRQoL between patients and physicians, representing an effective approach to improving the quality of care for patients. Here we review the importance of considering PROMs in decision-making for patients receiving first-line treatment for colorectal cancer. Making choices between treatments requires valid and reliable PROMs across clinical trials so that patients have the high-quality information needed to make patient-centered treatment decisions consistent with their personal preferences. In the era of precision oncology there is no doubt that a biomarker based approach impacts on the QoL of our patients, as for Pembrolizumab in MSI-H/dMMR mCRC patients, or by understanding and predicting resistance to treatments and, therefore, sparing unnecessary toxicity without improving outcomes, as for mCRC patients and the use of anti-EGFR treatments in tumors harboring mutations in the EGFR-pathway like *KRAS*, *NRAS* and *BRAF* mutations. In the last decade awareness about this topic has led to the implementation of some strategies, including guidelines, and founding support, that have increased the application of PROs in mCRC trials (**Table 1**). Even though, efforts are needed to give PROs their due if we want to practice real patient-centered medicine. For the moment, standard collection, analysis, and reporting of these measurements is not universal in clinical trials nor in usual clinical practice.

**Table 1:** Summary of key clinical trials in first line mCRC and their assessment of QOL.

Therapy	Disease setting	Trial	Survival outcomes	HR (95%CI)	QoL	Ref.
IFL-Bevacizumab	mCRC	AVF2107g Phase III NCT00109070	Placebo-IFL • Median OS: 15.6 m IFL-Bevacizumab • OS gain: 4.7 m	OS: 0.66 (0.54-0.81)	No QoL benefit	(65, 66)
CAPOX or FOLFOX4 – Bevacizumab	mCRC	NO16966 Phase III NCT00069095	CAPOX or FOLFOX4 • Median PFS: 8.0 m • Median OS: 19.9 m CAPOX or FOLFOX4-Bevacizumab • PFS gain: 1.4 m • OS gain: 1.4 m	PFS: 0.83 (0.72-0.95) OS: 0.89 (0.76 -1.03) NS	QoL not assessed	(67)
FOLFIRI-Cetuximab	EGFR-expressing, RAS wild-type mCRC	CRYSTAL Phase III NCT00154102	FOLFIRI • Median PFS: 8.4 m • Median OS: 20.2 m FOLFIRI-Cetuximab • PFS gain: 3 m • OS gain: 8.2 m	PFS: 0.56 (0.41-0.76) OS: 0.69 (0.54-0.88)	QoL a secondary endpoint	(8, 68)
FOLFOXIRI-Bevacizumab	mCRC	TRIBE Phase III NCT00719797	FOLFIRI-Bevacizumab • Median PFS: 9.7 m • Median OS: 25.8 m FOLFOXIRI-Bevacizumab • PFS gain: 2.4 m • OS gain: 4.0 m	PFS: 0.75 (0.62-0.90) OS: 0.80 (0.65-0.98)	QoL not assessed	(7)
FOLFOX4-panitumumab	Wild-type RAS mCRC	PRIME Phase III NCT00364013	FOLFOX4 • Median PFS: 9.2 m • Median OS: 20.9 m FOLFOX4-Panitumumab • PFS gain: 1.6 m • OS gain: 7.4 m	PFS: 0.68 (0.54-0.87) OS: 0.74 (0.57-0.96)	QoL a tertiary endpoint	(67, 69)
Pembrolizumab	First line treatment of MSI-H or dMMR mCRC	KEYNOTE177 Phase III NCT02563002	5-FUbased ChT ± bevacizumab/cetuximab • Median PFS: 8.2 m Pembrolizumab • PFS gain: 8.3 m	PFS: 0.59 (0.45-0.79) OS: 0.74 (0.53-1.03) NS	QoL exploratory endpoint	(10, 70)

#### 4.1.1 Capecitabine vs. 5-Fluorouracil

5-FU is the key to mCRC treatment. Intravenous (IV) 5-FU or oral capecitabine have been tested in combination for most first- and second-line strategies. Both Capecitabine and IV 5-FU are considered equivalent (71). Patients tend to prefer oral chemotherapy treatment as IV treatment can have a negative impact on patient QoL as it is associated with longer hospital stays, discomfort, psychological stress, and complications associated with catheters (such as thrombosis and infection). However, some trials also showed a significantly better QoL (using not validated questionnaires) of certain regimens of IV 5-FU compared to capecitabine, probably because of reduced toxicity, reinforcing the concept that patient decision is strongly affected by toxicity profile and not convenience (72, 73).

#### 4.1.2 Oxaliplatin vs. Irinotecan

FOLFOX and FOLFIRI are considered equally effective, and the choice of first line can be guided by their different toxicity profiles. When comparing QoL, FOLIRI-Bevacizumab has better outcomes on the FACT-C and FACT/GOG-Ntx scales than FOLFOX-Bevacizumab (74).

Capecitabine is more frequently combined with oxaliplatin (CAPOX) and less frequently with irinotecan (CAPIRI), as it has higher adverse events than FOLFIRI, even though a dose-modified CAPIRI schedule could overcome this difference (75). This combination has not been directly compared in terms of QoL, but a retrospective analysis of the CAIRO and CAIRO2 trials evaluated the percentage of patients having a 10 point increase in global QoL measured by EORTC-30 questionnaires between the groups of more than 75 years, 70–75 years, and less than 70 years. The results did not find statistically significant differences between groups, with CAPIRI showing a 10-point increase in 25% vs. 5% vs. 21%, respectively, p = 0.248, and for CAPOX-Bevacizumab, a 33% vs. 15% vs. 17%, respectively, p = 0.588 (76). Surprisingly, in both treatments, the patients with the highest percentage of 10-point increases in QoL were the group of more than 75 years, even though they had the worst survival and discontinued more often due to toxicity instead of progression compared to younger patients. This opens the question of how these data were analyzed or if there could be some bias that explains these results.

Even though triplet FOLFOXIRI has been evaluated in modern studies, it draws attention to the fact that neither the TRIBE nor the TRIBE2 (7, 77) studies have evaluated QoL relative to doublet chemotherapy, representing a clear limitation of both studies, especially when the triplet combination treatment has a higher risk of toxicity that has to be taken into account (69, 78).

#### 4.1.3 Anti-VEGF therapy

There are few available data about the direct impact of the addition of Bevacizumab to chemotherapy in terms of QoL. Only the AVF2107 phase III trial comparing 5FU, leucovorin and irinotecan with or without Bevacizumab reported no statistical differences in the deterioration of QoL reported by various questionnaires, including FACT-C total score and by MID (66). None of the other pivotal trials combining first line chemotherapy with Bevacizumab reported QoL outcomes (67, 79). Bevacizumab it is generally well tolerated, and the decision on whether to add Bevacizumab or not should depend on the toxicity profile and individual risk of the patient. Capecitabine plus bevacizumab has been shown to be effective and safe in elderly patients and represents a good treatment option in this population (79).

#### 4.1.4 Anti-EGFR therapy

KRAS and BRAF wild-type left-sided tumors benefit in terms of OS from the addition of cetuximab or panitumumab to FOLFOX or FOLFIRI chemotherapy (8, 9). There is a lack of literature comparing cetuximab and panitumumab, but the different toxicity profiles of both treatments could be helpful in selecting the most appropriate treatment for our patients. Higher rates of grade 3/4 skin toxicities, hypomagnesemia, fatal adverse events, grade 3/4 neurotoxicity (likely from oxaliplatin), and treatment stopping were linked to Panitumumab, while higher rates of skin rash, infusion reactions, and gastrointestinal toxicity were linked to Cetuximab (80). No deterioration in HRQoL measured by EORTC-QLQ-30 in the cetuximab plus chemotherapy arm was reported in the CRYSTAL study. Interestingly, social functioning did not differ between arms, a point at which skin reactions could have reflected impaired outcomes in QoL (68). Early tumor shrinkage was related to better improvements in HRQoL in the single arm phase II QUACK measured by EORTC QLQ-C30 in patients treated with chemotherapy plus cetuximab (81).

Panitumumab has also been evaluated in terms of HRQoL by means of EQ-5D questionnaires (which are a less objective and standardizable scale) in a retrospective analysis of the PRIME trial, that evaluated the quality-adjusted time without symptoms of disease or toxicity of treatment, resulting in 2.3 ± 1.0 (SE) additional quality-adjusted months (P < 0.03) (82).

Unfortunately, the use of Capecitabine combined with anti-EGFR is not recommended due to the increase in toxicity, leading to a dose reduction or discontinuation of the chemotherapy schedule (83). In frail or elderly patients, monotherapy with anti-EGFR monoclonal antibodies may be a good option for disease control, as no data about QoL were provided in the study (84).

Notably, none of the trials evaluating anti-EGFR therapy in mCRC used dermatological QoL measures such as the Dermatology Life Quality Index (DLQI), which could help mirror the real effect of skin toxicity on patients’ QoL.

#### 4.1.5 Maintenance therapy

In pivotal trials of chemotherapy doublets, treatment continuation was scheduled until disease progression or unacceptable toxic effects (8, 85–87). As a result, the cumulative neurotoxicity caused by oxaliplatin’s decreased QoL (88, 89) has led to the development of maintenance or intermittent strategies without impairing survival outcomes (90). Hence, maintenance therapy with fluoropyrimidines and a monoclonal antibody after induction with oxaliplatin chemotherapy must be considered and discussed with every patient. Even though studies of maintenance strategies vs. a complete chemotherapy-free interval have shown contradictory results, most of the studies corroborate better disease control and progression-free survival without a clear benefit to overall survival (90, 91). This important reason justifies a complete evaluation of HRQoL and patients’ preferences when deciding to perform maintenance treatment.

The CAIRO3 trial that evaluated HRQoL by EORTC QLQ-C30 of Capecitabine plus Bevacizumab maintenance treatment vs. observation. During maintenance treatment, global quality of life (mean change 0.03, 95% CI - 0.35 to 0.41), functioning, and symptom subscales of QoL did not deteriorate. In the observation arm, small but significant improvements were found for QoL (1.4, 95% CI - 0.8–2.1) and four functioning subscales. Between the two arms, there was a between-group difference in global QoL of 4.2 points (95% CI 1.5–6.8), which is well below the threshold of ten points that is deemed to be clinically relevant (91). Consistently, in the AIO 0207 trial comparing observation to maintenance treatment with bevacizumab alone or with 5-FU/LV, the maintenance strategy did not hurt QoL compared to observation, as measured by the EROTC QLQC-30 and QLQ-CR29 questionnaires, and there were no significant differences between the two maintenance arms (92). The FOCUS4 trial evaluating maintenance therapy with capecitabine shows similar results without proving a detrimental effect on HRQoL by the EORTC QLQ-C30 and QLQ-CR29 questionnaires (93). It has to be said that all the patients enrolled in these trials were randomized after the induction treatment, representing a possible bias when interpreting the data, as the impact of induction therapy on patients’ QoL is not evaluated, and patients who do not achieve disease control after the induction phase will be ineligible for maintenance trials. Bevacizumab as a single agent has no therapeutic value and is therefore not recommended (94, 95).

Concerning panitumumab, the VALENTINO study with panitumumab monotherapy vs. panitumumab plus 5FU/LV maintenance after induction therapy found an overall recovery of health-related QoL, as well as the expected prevention of oxaliplatin-related neurotoxicity, as measured by the EORTC QLQ-C30, QLQ-CR29, and EuroQol EQ-5D (96). Therefore, the maintenance strategy with 5FU/LV plus panitumumab did not significantly impair patients’ QoL with an improvement in PFS. In the same direction, the PANAMA trial draws the same conclusions (96).

Data on cetuximab maintenance and QoL are lacking, as there are no published data from the COIN-B trial, the MACKRO2 trial, or the NORDIC-7.5 study. None of these trials evaluating maintenance with cetuximab were combined with fluoropyrimidines. Actual guidelines recommend a maintenance strategy with combined fluoropyrimidines plus anti-EGFR treatment.

#### 4.1.6 Immunotherapy

The KEYNOTE-177 evaluates HRQoL as an exploratory endpoint using the EORTC QLQ-C30 and QLQ-CR29 questionaries and the EQ-5D-3L visual analog scale and health utility scores. Interestingly, the authors have dedicated an exclusive publication to reviewing this topic, supporting the community’s growing concern about the need for such endpoints in clinical trials. Pembrolizumab showed clinically and statistically meaningful improvements in HRQoL compared with chemotherapy in patients with previously untreated MSI-H or dMMR mCRC from baseline to pre-specified week 18, including time to treatment deterioration, physical functioning, social functioning, and fatigue (70).

### 4.2 First line clinical trials

Particular cases with known specific molecular alterations may benefit from considering a clinical trial with the addition of targeted therapy, as some interesting trials in the first line setting are currently ongoing, such as BRAFV600E, HER2, etc. Hopefully the evolution of this patient population in terms of QoL will be properly documented and reported, as characterization of this patient’s journey will be helpful for clinicians to understand how this selected population evolves over time in terms of QoL, an important question that the whole community needs to answer, including regulatory agencies. Patients with a clinical evolution that can reveal an underlying biology with special molecular features may benefit from additional tests. Even though clinical guidelines in the first line setting only recommend testing for microsatellite instability or mismatch repair deficiency (MSI-H, MSS, dMMR, and pMMR), BRAF V600E mutation status, RAS mutation status, and HER2 amplification, they do not routinely recommend performing Next Generation Sequencing (NGS) in mCRC patients (61, 62), a systematic review suggests that colorectal cancers could benefit from molecular profiling by NGS and Molecular Tumor Board (MTB) assessment (97). Complex molecular profiles, which encourage resistance to systemic agents and targeted therapies, place restrictions on the treatment of mCRC; therefore, if practical, NGS can be useful for academic research or prescreening for clinical trials. MTB can be performed in certain academic centers and can provide standard-of-care or experimental therapeutic recommendations for most patients. This approach could be fundamental in the treatment strategy; some reports state that patients treated with targeted therapy following MTB recommendations had better outcomes, even though limited drug access is the major concern and limitation. Still, MTBs could help improve access by keeping track of relevant clinical trials and by registering MTB recommendation outcomes, serving as a forum to keep oncologists and multidisciplinary teams updated (98).

## 5 Discussion

The patient journey of mCRC patients is a complex and challenging experience that starts with diagnosis and requires the collaboration of multiple healthcare providers. The experience and perception of the patient at every step are what guide the patient’s journey. This approach can be measured by PRO questionnaires, but sadly, their implementation in daily use or even clinical trials has proved to be low.

There’s a clear impact of clinical and translational research on the patient journey, as clearly reflected in the overall survival improvement of mCRC in the last decade, but each individual patient will have a different experience about it, which could be followed along the subsequent treatment lines by a strategy of patient-journey studies. This research model could help researchers and the community clearly identify the needs and concerns of patients in critical moments. There are several figures involved in the journey of mCRC who should work as a multidisciplinary team to practice patient-centered care.

Decisions about treatment for patients with mCRC require consideration of the effects of both the disease and its treatment on QoL, in addition to treatment efficacy. The median survival time for patients with mCRC has increased substantially over the last decade, but unfortunately, only <26% of patients live longer than 5 years. Because of this, consideration of QoL is critical, especially in elderly asymptomatic or minimally symptomatic patients, where trade-offs between the lengthened survival and its possible detrimental effects on QoL must be considered.

It is important to note that the impact of mCRC and its treatment on QoL can vary greatly among individuals. Factors such as age, overall health, and personal preferences can influence how patients perceive the trade-offs between survival and QoL. Therefore, a comprehensive assessment of both medical and patient-reported outcomes is necessary to make informed treatment decisions. In the last decade, with the arising precision medicine paradigm, a shift towards a patient-centered approach has been done, with interesting data coming from biomarkers guided clinical trials, some of them accompanied by improvements in QoL. Disappointingly, in many occasions there is still a lack of clinically meaningful data about QoL in many of the already published trials for first-line treatment. The questionnaires used are often general ones if they are performed and sometimes interpretation may be challenging. Information about HRQoL could be improved by a better selection of PROMs and a more extended analysis of the data. HRQoL and PRO are a growing need as estimands in clinical trials, especially in the upcoming years when we will probably witness a revolution in precision medicine and new approvals of targeted drugs and immunotherapy. Incorporating HRQoL and PRO measures in clinical trials is crucial to gaining a comprehensive understanding of the patient experience and treatment outcomes. Additionally, PRO measures can aid in identifying potential side effects or adverse events that conventional clinical endpoints might not be able to capture. By utilizing disease-specific questionnaires and conducting thorough analyses, researchers can provide valuable insights into the impact of new therapies on patients’ quality of life. This will not only enhance the evaluation of treatment efficacy but also contribute to informed decision-making in the evolving landscape of precision medicine and emerging therapies. Ultimately, this comprehensive understanding of patient experience and treatment outcomes can inform regulatory decisions and guide clinical practice for the benefit of patients.

## Author contributions

MC: Writing – original draft, Writing – review & editing. IB: Writing – review & editing, Supervision. JR: Writing – review & editing. NS: Writing – review & editing. FS: Writing – review & editing. AG: Writing – review & editing. AA: Writing – review & editing. JT: Writing – review & editing. EE: Supervision, Writing – review & editing.
